# News and Notes

**Published:** 1906-11

**Authors:** 


### NEWS AND NOTES.

APPLICANTS wanted for position of Junior House Surgeon at Wesley Memorial Hospital, Atlanta, Ga.

St. LOUIS *Medical and Surgical Journal* has combined with the *Medical Mirror*. The former journal is absorbed in the latter.

GREAT efforts are being made in certain quarters of London to obtain legislation for the prevention of smoking amongst boys.

DR. RHETT GOODE, of Mobile, has been elected dean of the Medical College of Alabama. He was graduated from this school in 1871.

THE Kansas City board of health has had printed a yellow card, to be posted in prominent places in restaurants where unsatisfactory sanitary conditions are found.

DR. NICHOLAS SENN, who has returned from his travels in Africa, states that, from an ethnological standpoint, he believes the African is nearly an unmixed Hamitic race.

DR. D. S. ARNOLD died September 27th at his apartments in the Peck building, Atlanta. Dr. Arnold was a well-known dentist, having come to the city many years ago from Alabama.

COMMITTEES have been organized in England and Germany to solicit subscriptions toward a fund to be presented to the wife of the discoverer of the spirochæta pallida, Fritze Schaudinn.

THE St. Louis Medical Society dedicated its new medical auditorium Saturday September 15. This progressive society has set an example that should be followed by many others throughout the country.

---

THE king of the Belgians, as sovereign of the Congo Free State, is offering a prize of 200,000 francs, open to all nationalities, for the discovery of a method of successfully treating sleeping sickness.

---

DR. S. G. PINCKNEY died at the home of his mother, Homestead, Grand-View-on-the-Hudson, September 21. Dr. Pinckney was well-known throughout the State as a specialist in nervous disorders. He is survived by his wife and one child.

---

PROFESSOR POZZI, of Paris, has been presented with a gold medallion portrait by the sculptor, Chaplain, and a memorial volume of researches by his former students on the occasion of the twentieth anniversary of his work at the Broca hospital.

---

THE Fifth District Medical Society met in Atlanta October 16. A banquet at the Piedmont followed the business and scientific program. Much credit is due Dr. E. C. Davis for the commendable work he is doing in organizing the medical profession of this district.

---

AT the meeting of the Association of Military Surgeons, held in Buffalo recently, it was announced that the Enno Sanders prize had been awarded to Major Pilcher for an essay on "The Training of the Medical Officer of the State Forces to Best Qualify him for Local Service and for Mobilization with National Troops."

KING EDWARD thinks the spread of sleeping sickness in Africa is one of international importance and has asked the Liverpool School of Tropical Medicine to submit a plan for the prevention of the disease. He promised his co-operation if it is in any way possible or practicable.

---

A SENSIBLE view of medical etiquette is held by the London *Spectator* which declares that, instead of being kept up, as people so often imagine, in the interests of physicians it is really maintained in the interests of the public. It is the public, not the doctors, who would suffer most, were medical ethics entirely done away with.

---

AT a meeting of the Brooklyn Society for Neurology, Dr. Walter G. Chase, of Boston, delivered an illustrated lecture on "Insanity and Nervous Diseases," illustrated by moving pictures. In order to obtain these Dr. Chase went, for several weeks, daily into an open field with a band of epileptics, stripped nude, but protected from the sun by blankets, and there waited for the convulsions to occur. The result was that hundreds of seizures were photographed.

---

THE Simon Fund of $25,000 for the furtherance of research on syphilis has been divided between Professor Neisser, of Breslau, who receives $20,000, and Dr. J. Siegel, who receives $5,000. Dr. Lesser has been awarded $1,500. The trustees of the Pettenkofer Fund, at a meeting recently held in Munich, unanimously awarded the prize founded in memory of the great hygienist to Dr. Schaudinn for his discovery of the parasite of syphilis. The amount has been paid to Dr. Schaudinn's widow.

---

THE Tri-State Medical Society of Georgia, Tennessee and Alabama has been merged into the Southern Medical Association. This was done with the adoption of by-laws and resolutions and the election of the following officers: President, Dr. H. H. Martin, Savannah, Ga.; first vice-president, Dr. Mack Rogers, Birmingham, Ala.; second vice-president, Dr. J. B. Cowan, Tullahoma, Tenn.; third vice-president, Dr. J. B. Tackert, Meridian, Miss.; secretary, Dr. Raymond Wallace, Chattanooga, Tenn.; treasurer, Dr. Y. L. Abernathy, Chattanooga.

---

AT the meeting of the Association of Military Surgeons of the United States, September 11-14, 1906, Buffalo, N. Y., the following officers were elected: President, Col. Valery Havard, assistant surgeon-general, U. S. Army; vice-presidents, Rear Admiral Presley M. Rixey, surgeon-general U. S. Navy; Col. George Tully Vaughan, late U. S. P. H. and M. A. Service, and Col. Joseph K. Weaver, N. G. Pa., Norristown, Pa.; secretary, Major James Evelyn Pilcher, U. S. A., Carlisle, Pa. (re-elected); assistant secretary, Capt. J. Carlisle De Vries, N. G. N. Y.; treasurer, Major Herbert A. Arnold, N. G. Pa. (re-elected).

---

THE sixth annual meeting of the board of governors of the Georgia Pasteur Institute was held in Atlanta, October 5th. Dr. Henry R. Slack, president, read his report for the year, which showed that they had treated 471 patients and of these only two have died. Three developed the disease after treatment was begun, but this was no fault of the treatment, as sufficient time had not elapsed to secure immunity. These patients have come from every Southern State, except Virginia and Kentucky. The following officers were elected for the ensuing year: President, Dr. Henry R. Slack, LaGrange; vice-president, Dr. G. H. McDuffie, Columbus; physician in charge, Dr. Jas. N. Brawner, Atlanta; assistant, Dr. E. C. Cartlige, Atlanta; pathologist, Dr. Claude A. Smith, Atlanta.

